# A university map of course knowledge

**DOI:** 10.1371/journal.pone.0233207

**Published:** 2020-09-30

**Authors:** Zachary A. Pardos, Andrew Joo Hun Nam

**Affiliations:** 1 Graduate School of Education, University of California, Berkeley, California, United States of America; 2 Department of Psychology, Stanford University, Stanford, California, United States of America; IBM Research, UNITED STATES

## Abstract

Knowledge representation has gained in relevance as data from the ubiquitous digitization of behaviors amass and academia and industry seek methods to understand and reason about the information they encode. Success in this pursuit has emerged with data from natural language, where skip-grams and other linear connectionist models of distributed representation have surfaced scrutable relational structures which have also served as artifacts of anthropological interest. Natural language is, however, only a fraction of the big data deluge. Here we show that latent semantic structure can be informed by behavioral data and that domain knowledge can be extracted from this structure through visualization and a novel mapping of the text descriptions of elements onto this behaviorally informed representation. In this study, we use the course enrollment histories of 124,000 students at a public university to learn vector representations of its courses. From these course selection informed representations, a notable 88% of course attribute information was recovered, as well as 40% of course relationships constructed from prior domain knowledge and evaluated by analogy (e.g., Math 1B is to Honors Math 1B as Physics 7B is to Honors Physics 7B). To aid in interpretation of the learned structure, we create a semantic interpolation, translating course vectors to a bag-of-words of their respective catalog descriptions via regression. We find that representations learned from enrollment histories resolved courses to a level of semantic fidelity exceeding that of their catalog descriptions, revealing nuanced content differences between similar courses, as well as accurately describing departments the dataset had no course descriptions for. We end with a discussion of the possible mechanisms by which this semantic structure may be informed and implications for the nascent research and practice of data science.

## Introduction

The emergence of data science [1] and the application of word vector models for representation learning [2–4] have, together, focused attention on surfacing structure from big data in ways that are scrutable and show signs of being able to contribute to domain knowledge [5, 6]. These neural models, stemming from cognitive theories of distributed representation [32], have been shown to encode a surprising portion of linguistic relationships learned directly from text [7]. They contribute to part of a quickly growing field around computational text and natural language modeling. While much of the recent focus of the field has centered on advancements in deep models for text generation and translation [8, 9], a separate thread of research has worked to explore and inspect the semantics and lexical relationships that can be surfaced, favoring simpler linear neural models for their interpretability and scrutability [10–13]. It is in the vein of this thread of research, and forwarding the general interest of data science to make meaning from observational data, that we conduct our study generalizing the application of computational text methods to a dataset of behaviors outside of the domain of language.

An embedding, or neural representation learned from sequential data, can be framed as an informational artifact mapping elements in a sequence to parts of a semantic structure formed by element contexts in the sequence [14–16]. In our study of the topical regularities of this semantic structure, the elements are courses appearing in the historic enrollment sequences of tens of thousands of students. Learning a course embedding, constructed from these sequences of course IDs, is learning a mapping of courses onto a semantic space informed by students’ preferences, their knowledge of courses at the time of enrollment, and the relationship of those courses to the curricular structure of degrees. Using this embedding, we highlight the breadth of information that can be communicated by student course selections using a model of distributed representation applied to a dataset of modest size. In addition to validating the model for what propositional domain knowledge it has encoded, we provide opportunities for additional topical regularities to surface through visualization and a mapping of the abstract course vector space onto a natural language space.

Our work relates to the field of learning analytics, where historical enrollment data have been used to predict the next courses a student will take [17, 18], the grade they may receive in those courses [19–21], and the prerequisites that may prepare them to achieve their goals [20, 22]. We contribute methods for learning the underlying semantics of curricular resources from data, a phase outlined in an early learning analytics vision document [23] as coming after predictive modeling and preceding adaptive course sequencing.

The workflow of our methodology is as follows: (1) process enrollment data into chronological sequences grouped by student (2) learn several neural embeddings of courses using different hyperparameter sets (3) draw on domain knowledge to create propositions to validate the embeddings against (4) conduct model selection based on validation scores (5) explore the relationships between courses, visually and algebraically (6) investigate the semantics of sparse areas of the course vector space by mapping them onto a space of course catalog descriptions and interpolating.

## Data, models, and optimization

**Fig 1:**
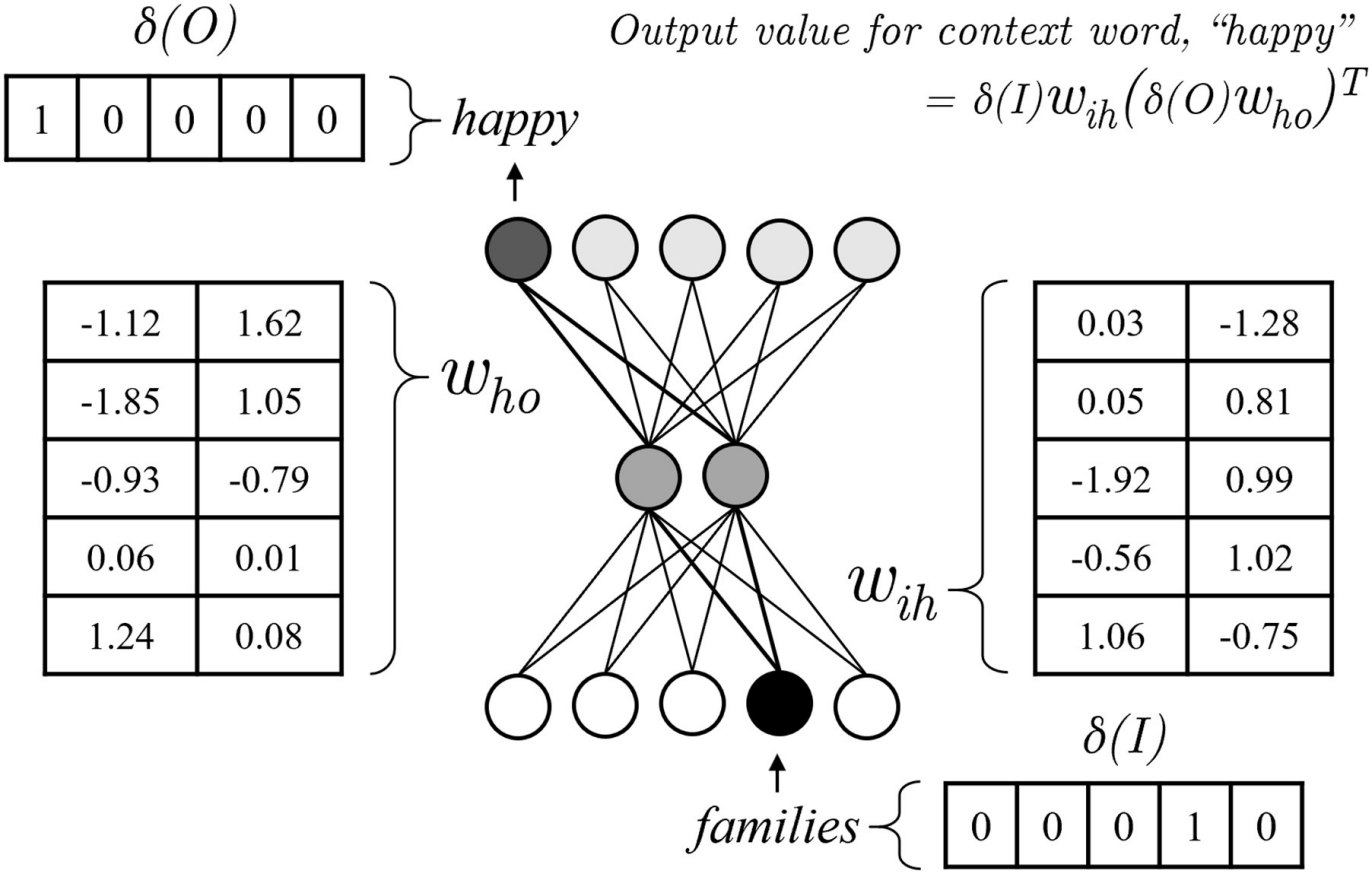
Skip-gram model. The three-layer neural network architecture of a skip-gram model. An example input word, *families*, is depicted with a one-hot representation as well as example weight coefficient matrices used to produce an output value prediction for the word *happy*.

Originally conceived of for natural language, the skip-gram and continuous bag-of-words (CBOW) models embed words into a high-dimensional vector space, with model weights adjusted through backpropagation to predict word contexts across a corpus. They can be posed as a three-layer neural network (Fig 1), similar in objective to an autoencoder [24], creating a lower dimensional representation of the input in the hidden layer by attempting to re-construct it in the output. Unlike autoencoders, skip-grams process a single input word (*I*) at a time (*t*) and capture chronology by predicting only *c* number of words to the left and right of the input word in calculating the loss (Eq 1). The intuition is that the meaning of a word can be inferred from the contexts in which it has been used and that those contexts can be summarized using neural networks.

$$\begin{array}{c} loss=-\sum_{d\in D}\frac{1}{\left|d\right|}\sum_{t=1}^{\left|d\right|}\sum_{t-c\leq j\leq t+c,j\neq t}\text{log}p\left(d_{t+j}|d_{t}\right) \end{array}$$ (1)

$$\begin{array}{c} p\left(O|I\right)=\frac{\text{exp}(\delta \left(I\right)w_{ih}{\left(\delta \left(O\right)w_{ho}\right)}^{T})}{\sum_{v\in V}\text{exp}\left(\delta \left(I\right)w_{ih}{\left(\delta \left(v\right)w_{ho}\right)}^{T}\right)} \end{array}$$ (2)

The objective function of the model (Eq 1) is to increase the predicted probabilities of the words within the context (*d*_*t*±*j*_) of the input word, *d*_*t*_. The size of the context window is determined by *j*, a hyperparameter of the model. Every document in a corpus (*d* ∈ *D*) is used for training, with each word at each position of the document serving as the input word once per training iteration, or epoch. For example, given the sentence, “*All happy families are alike*” Fig 1 shows how a skip-gram might predict the context words given *families* as the input word. The input word is first processed into one-hot form, denoted by the *δ* notation. This one-hot vector, which is as long as the total number of unique words used in the corpus, is multiplied by the weight coefficient matrix (*w*_*ih*_) representing the edge weights between the input layer nodes and the hidden layer nodes. The result is a vector of length equal to the number of hidden layer nodes. This vector length is a hyperparameter of the model. The vector is then multiplied by the weight coefficient matrix (*w*_*ho*_) representing the edge weights between the hidden later and the output layer. The output layer contains the same number of nodes as the input layer. Given that the word *happy* appears in context with the input word, it will be included in the loss calculation. The higher the output value of the context word, converted to a probability via the softmax function (Eq 2), the lower the loss. Both weight matrices are trained to minimize the loss through stochastic gradient descent. If the one-hot input layer of the model were directly connected to the one-hot output layer forming a multinomial logistic regression, the weights (*w*_*io*_) would simply be the distribution of target words across all the input word’s contexts. The insertion of a hidden layer of a lower dimensionality than the input and output layers adds a layer of shared featurization in which regularities, or patterns, must be captured in order to reduce the loss. The input-to-hidden-layer edge weights (*w*_*ih*_), after training, yield the continuous vector representations of the words, the collection of which is an embedding. The lack of non-linear activations in this model, unlike a deep network, imparts the embedding with the properties of a vector space. These properties allow for semantics to be retained after arithmetic and scalar manipulation of word vectors in this space. We apply this modelling approach to the extra-linguistic sequences of course enrollments from each student’s course history, as opposed to its traditional application to the sequences of words from documents.

We used student enrollment data from UC Berkeley, a public research university in the University of California system. The data spanned from Fall 2008 through Spring 2016 for a total of 23 semesters including summer sessions, with 2,129,810 class enrollments made by 124,203 anonymized undergraduate students in 163 degree programs. Considering courses that undergraduates enrolled in, graduate courses included, there were 7,997 unique lecture courses across 197 subject areas. A subject area is the most granular category of academic unit at UC Berkeley, followed by department, division, and college. Professional schools are standalone units but will be counted in analyses as both subjects and divisions.

Courses designated as “special topics” have not yet received an approved unique course number and instead use the same generic course number in each department. To differentiate between these courses, we appended the course identifier with the instructor’s name. The robustness of a skip-gram model can deteriorate when there are too few data points for elements in the vocabulary (i.e., course ID tokens). To reduce this source of noise, we filtered out courses that had less than 20 total enrollments in the eight year span of the dataset. Additionally, we removed enrollments for non-lecture credit, such as independent research and senior theses, decreasing the unique course IDs in the model from 7,997 to 4,349.

We encoded each course taken by a student as a one-hot, allowing an undergraduate career to be represented as a sequence, *S*, of one-hots, serializing courses taken at the same time by randomizing their within-semester order. Every occurrence of a course in every student’s enrollment sequence represents a training instance, with the prediction targets being the courses in the enrollment sequence prior to and after the input course within a specified window (*d*_*t*±*j*_). After training, the input-to-hidden-layer edge weights (*w*_*ih*_) yield the continuous vector representations of courses. Our open-source tool, used for learning course representations and interactive visual exploration, can be found online [25].

This collection of course vectors, and the relationships they may encode, is the component of interest, as opposed to the model’s predictions of the courses in context. It is necessary, therefore, to tune hyperparameters of the model to maximize the validity of the encoded relationships as opposed to maximizing its accuracy in predicting courses in context. In word representation learning, a sampling of propositions in the broad categories of semantic and syntactic word relationships are hand defined and serve as the set of ground truth relationships with which the embedding can be validated against [2]. Given our novel application to university enrollment data, finding sources of validation in this domain was a challenge.

One source of validation we identified was the set of cross-listed courses, which are courses with multiple listings in different departments (e.g., *Economics* C175 and *Demography* C175 correspond to the same course). If two course listings were not explicitly cross-listed with each other but were cross-listed with a shared third course, we considered the set of three to be cross-listed. This produced a validation set of 1,472 cross-listed pairs enumerated from 443 cross-listed sets of two or more courses.

For a second validation set, we collected sets of credit equivalent courses. These 128 sets consisted of 250 courses across 48 subjects. Courses within these sets were considered so similar in content to one another by faculty that the Office of the Registrar will not give a student credit for taking both (e.g., Linear Algebra is credit equivalent or credit “restricted” with Honors Linear Algebra). The data were collected from a course information system website of the University and manually parsed. We chose to treat all levels of equivalency (full, partial, conditional) the same, assuming all equivalencies exhibited enough conceptual similarity to be of use in validating the models. We applied the transitive property, that two courses would be considered equivalent if they shared a third course that was equivalent with the two. This set distinguishes itself from the cross-listed pairs in that the courses in those pairs are the same, not just equivalent. This produced a validation set of 381 credit-equivalent pairs enumerated from 128 credit-equivalent sets of two or more courses.

We optimized choice of model architecture (skip-gram vs CBOW) and six hyperparameters of the representation learning model: window size (1 to 32), vector size (2 to 300), the use of hierarchical softmax, the use of negative sampling, the number of noise words drawn for negative sampling, and the threshold for down-sampling higher-frequency words. Using the cross-listed course pairs and the credit equivalency pairs, we queried the models for the nearest neighbors of each course in the pair, taking the rank of the expected course for each, then taking the median rank across all pairs (Algorithm S1). Because optimizing by a different metrics could yield a different top model, we allowed the optimization metric to be another point of comparison. The nearest neighbor rank of one course in the validation pair to the other was determined using cosine similarity, using the median rank across pairs in a validation set as the error metric for that set, performed both ways for each pair since the mutuality of nearest neighbors is not assumed. This would be comparable to performing model selection of language models by choosing the model with the highest median similarity based on pairs of synonymous words.

**Algorithm S1** Validation Score

1: **procedure** Validation Score(*validation*_*set*)

2:  medians ← new list

3:  **for** ∀*sets* ∈ *validation*_*set* **do**

4:   scores ← new list

5:   **for** ∀ course *pc* ∈ *s* **do**

6:    **for** ∀ course *c*′ ∈ *s*, *c* ≠ *c*′ **do**

7:     scores.add(rank of *c*′ using nearest neighbor to *c*)

8:   set_score ← median(scores)

9:   medians.add(set_score)

10:  *validation*_*score* ← *median*(*medians*)

11:  **return** validation_score

We experimented with treating cross-listed courses in one of two ways. The cross-listed sets of courses could be collapsed into a single course with only one distinct course ID in our enrollment data or they could be treated as individual courses belonging to the respective departments in which they were listed. The decision could have an impact on the resultant course vector space. An embedding based strictly on course content would place individually treated cross-listed courses (e.g., *Economics* C110 and *Political Science* C135) into the exact same point in a semantic space, however, this is unlikely to occur in practice since students majoring in Economics and Political Science tend to favor enrollment in the course listing that is within their home department. The difference in major distributions for each listing would change the course enrollment contexts of each, resulting in different learned vector representations. Alternatively, collapsing the cross-listed courses would force these courses to share all course enrollment histories. This could have the result of bringing many *Economics* and *Political Science* courses closer to one another since they would now have a mutual course in common. We tested which approach led to a better embedding by collapsing none, half, and all cross-listed courses in the enrollment data and comparing the performance of models trained on each version of the data. A model with no collapsed cross-listed courses would have the full set of cross-listed courses available to validate against while a model with all cross-listed courses collapsed would have none. To compare across the models optimized by different metrics and different cross-list collapse proportions, we held out 20% of the validation sets to serve as test sets.

**Fig 2:**
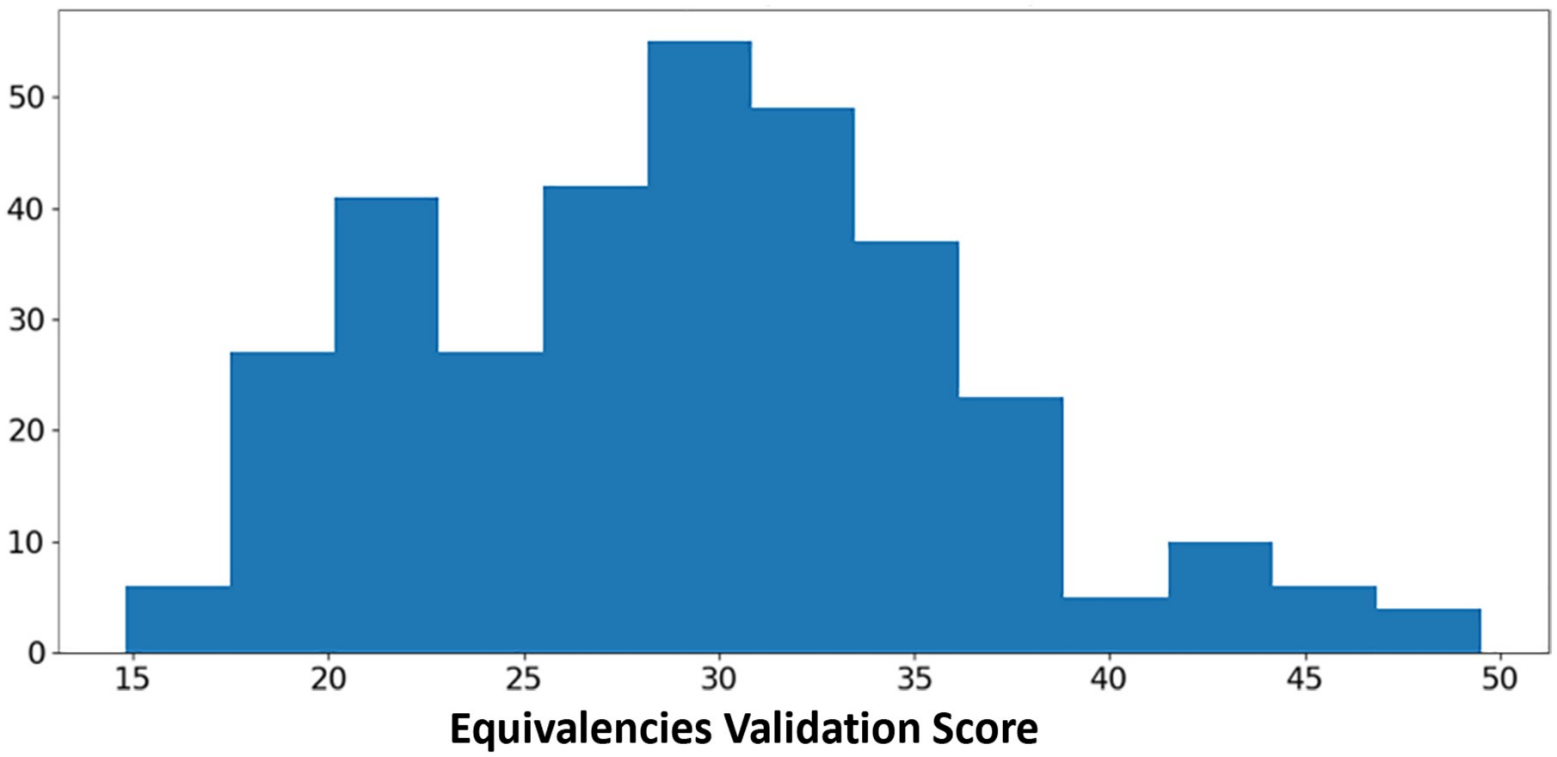
Histogram of course equivalency scores. Distribution of course equivalency validation scores from 400 model experiments, calculated as the median similarity rank of each pair. Lower is better. Scores > 50 omitted due to high skew.

We ran 400 models with different hyperparameters for each cross-list collapse experiment. The best performing model by overall validation score for each validation set and each cross-list percentage within that validation set is shown in Table 1 along with its respective scores on the test sets. The models trained with no collapsing of cross-listed courses produced the best overall test scores on both the equivalencies and cross-listings sets. Among those models, the best performing model on equivalencies also performed well on cross-listings; however, the opposite was not true. The distribution of the model equivalency validation scores for 0% cross-list collapse, from which the best model was selected, is shown in Fig 2.

**Table 1:** Best models by overall validation score (median rank) for each validation set and cross-list collapse percentage.

Model Selection Metric	Proportion of Collapsed Cross-listed Courses	Cross-lists Validation Score	Equivalencies Validation Score	Overall Validation Score	Cross-lists Test Score	Equivalencies Test Score	Overall Test Score
Cross-lists	0%	12	47.5	29.75	19.5	181	100.25
Cross-lists	40%	10	48.5	29.25	16.5	303.5	160
Cross-lists	100%		42	42		303.5	303.5
Equivalencies	0%	23.5	17	20.25	28	31.5	29.75
Equivalencies	40%	16.5	16.5	16.5	33	33	33
Equivalencies	100%		16	16		38	38

A skip-gram model (vector size = 229, window = 8, and negative sampling = 15, hierarchical softmax = 0, down-sampling threshold = 7.356e-4) performed best in minimizing the combined ranks of the two test sets and was *the* best model on the equivalencies test set. Students in our dataset took an average of four courses per semester, thus the empirically arrived at window size of eight is equivalent to two academic years of context. Selecting a model that exhibited generalizability within the equivalency and cross-listing task was important, as it would be used in subsequent exploratory analyses and to generalize to different tasks [26]. With a learned embedding in hand, optimized using relationships between a wide swath of courses across subjects, we proceed to scrutinize the embedding for other forms of topical and pedagogical regularity. Note that while we aimed to represent a wide variety of courses and disciplines at UC Berkeley in the following analyses, several comparisons and exemplars were chosen based on the authors’ personal domains of familiarity and input from several faculty subject matter expert colleagues.

## Analogy validation

We created an analogy validation set consisting of five course relationship categories to evaluate if the embedding encoded additional domain knowledge of courses. We sought to define course relationship categories which involved many departments on campus and which were as objective as possible in nature. The relationships between courses and their *honors* version and between courses and their *online* counterpart were two categories defined from superficial course number prefixes, while pairs of courses in a *sequence*, *mathematical rigor* pairs, and *topical* relationships were categories defined using first-hand institutional prior knowledge.

**Fig 3:**
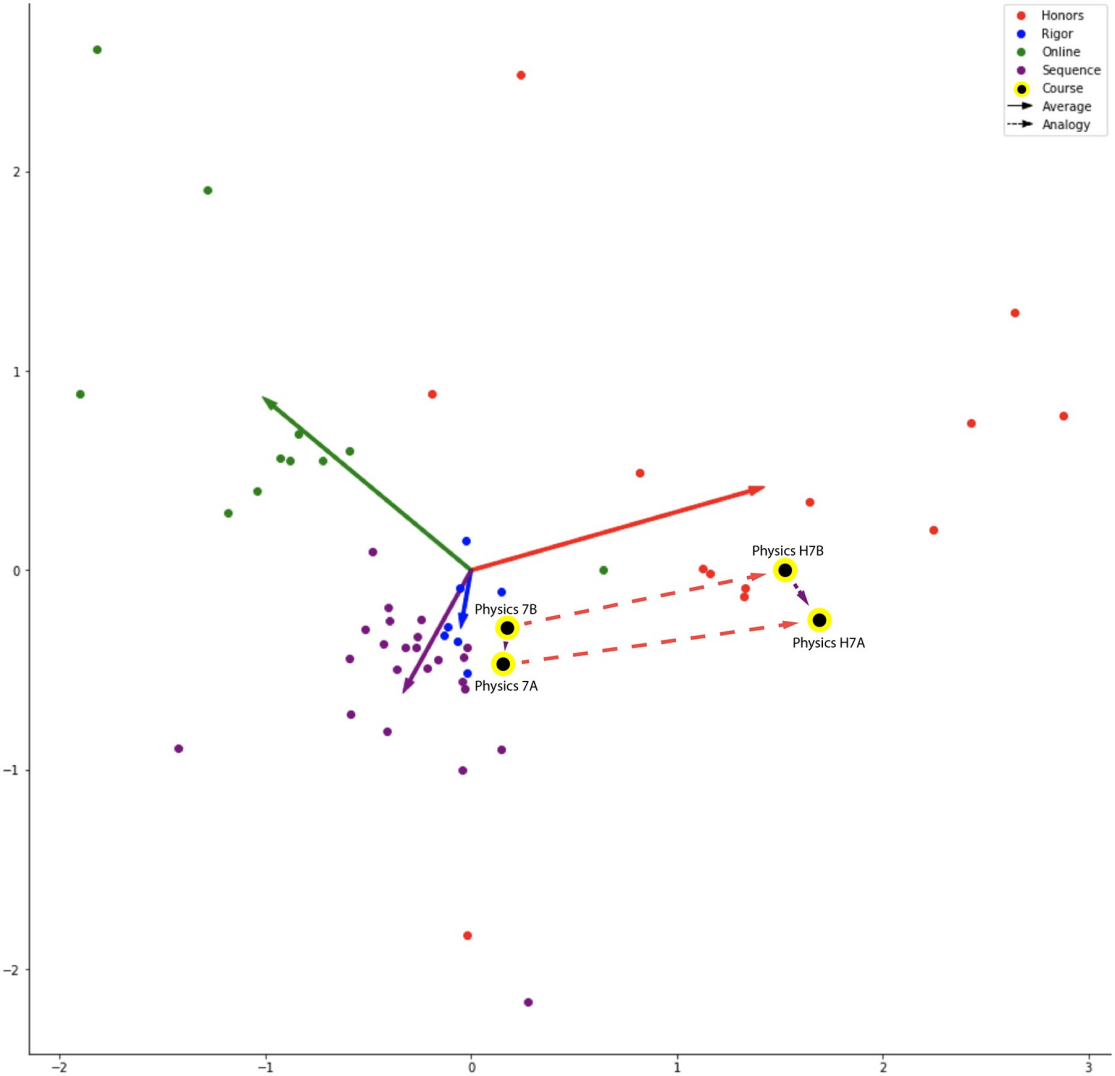
PCA analogy visualization. PCA of vector offsets with a Sequence and Honors constellation depicted using *Physics* courses.

Sequence relationships were between courses to be taken in adjacent semesters in a prescribed order. For example, *Mathematics* 1A followed by 1B in the next semester. *Physics* 7A and 7B follow the same pattern, which can form the analogical relationship, “*Mathematics* 1A is to *Mathematics* 1B as *Physics* 7A is to *Physics* 7B,” represented in vector arithmetic form as, “vec[*Mathematics* 1B]—vec[*Mathematics* 1A] + vec[*Physics* 7A] is most cosine similar to → vec[*Physics* 7B]” seen in Table 2 and visualized, in part, using PCA in Fig 3. In this approach, the representation of *Mathematics* 1A is removed from *Mathematics* 1B, leaving the vector offset representing the concept of sequence. This sequence vector is added to the *Physics* 7A vector, intending to yield a vector nearest to the target *Physics* 7B vector. The lower the nearest neighbor rank of the target course, the better the model has captured this relationship from isomorphisms in the vector space formed from patterns of enrollment behavior. To visualize the regularities in the space enabling analogy completion, we reduced the dimensionality of vector offsets (i.e., vec[Course A]—vec[Course B]) as well as the vectors of *Physics* 7A, *Physics* H7A, *Physics* 7B, and *Physics* H7B to two dimensions using PCA. We then visualized these points in Fig 3, which depicts the Sequence and Honors relationships, creating an imperfect formation of a parallelogram representing an analogy constellation from *Physics* courses.

**Table 2:** A selection of analogy results from each of the six relationship categories.

Relationship	Results (examples)
Honors	*Mathematics* H1B—*Mathematics* 1B + *Physics* 7B → *Physics* H7B
Online	*African American Studies* W111—*African American Studies* 111 + *Engineering* 7 → *Engineering* W7
Sequence	*Mathematics* 1B—*Mathematics* 1A + *Physics* 7A → *Physics* 7B
Mathematical Rigor	*Mathematics* H1B—*Mathematics* 1B + *Economics* 140 → *Economics* 141
Topical (with 2 subjects)	*Economics* C110 (game theory)—*Statistics* 155 (game theory) + *Statistics* 151A (linear modeling) → *Economics* 141 (linear modeling)
	*Psychology* 102 (computing)—*Psychology* 1 (introductory) + *Statistics* 134 (introductory) → *Statistics* H194A (honors seminar) [intended course was *Statistics* 133 (computing), rank 8]
Topical (with 3 subjects)	*Computer Science* 189 (machine learning)—*Statistics* 154 (machine learning) + *Statistics* 150 (random processes) → *Electrical Engineering* 126 (random processes)
	*History of Art* 34 (Chinese art)—*Chinese* 1A + *Japanese* 1A → *History of Art* 62 (Italian Renaissance art) [intended course was *History of Art* 35 (Japanese art), rank 2]

We defined the relationship category of mathematical rigor by identifying course pairs that shared content but utilized varying degrees of math. For example, while *Economics* 140 and 141 both cover Econometrics, 140 approaches it with a greater focus on principles with scalar operations whereas 141 uses rigorous proofs with linear algebra and probability theory.

The final relationship categoriy we coded was topical similarity between courses offered in two or three different subjects; *Statistics* 155 and *Economics* C110, for example, both cover game theory. The course relationship categories are listed in Table 2 in decreasing order of the prior domain knowledge expected to be held by students. Online and Honors courses are easily knowable from the coding syntax of the course number in the catalog. Sequence and rigor relationships, however, do not have consistent coding, but are communicated both formally by course descriptions and degree programs, and colloquially by peers and advisers. Sequences are often identifiable through suffixes (B course usually follows the A), but are sometimes less obvious, such as *Korean* 111 following *Korean* 102. Likewise, mathematical rigor (when not also an honors relationship) requires significant domain knowledge of the subject to identify. Cross-subject topical relationships are the most complex, requiring familiarity with the course offerings of two or three different subject areas. We might expect all students to understand the honors and online relationships, however, it is unlikely that any student not studying *Korean* would fully know the course sequences in that department, and a very limited number of students could connect courses across multiple disciplines by topic, making these relationship analogies a test of uncommon knowledge on courses and their relationships.

For all but the topical relationships, we tested the analogies in round-robin fashion (e.g., comparing one sequence pair with every other sequence pair and finding its rank for each). With 23 sequence pairs, 18 mathematical rigor pairs, 14 honors pairs, and 12 online pairs, we generated 1008, 576, 364, and 264 analogy equations respectively. Since topical relationships required two comparable course pairs and therefore lacked the fungibility of the other relationship categories, we generated 4 × *N* analogies using N = 11 quadruples for a total of 44 analogy equations. We considered the analogy completion to be successful if the rank 1 nearest neighbor was the anticipated target course to complete the analogy.

The accuracy of the course embedding in completing all 2,256 analogies generated from permutations of the 77 relationship pairs was 40%. The median rank of the intended target course in honors, online, sequence, mathematical rigor, and topical relationships were 4, 5, 1, 5, and 15, respectively, with accuracies (% rank 1) of 28.98%, 29.17%, 58.23%, 13.37%, and 17.5%. This overall accuracy rivals the 61% accuracy seen on syntactic and semantic lexical relation validations of word embeddings [2] which were trained on a dataset three orders of magnitude larger (1B words vs. 3.7M enrollments) with three times the average number of observations per element (1,400 per word vs. 462 per course). There are no results of greater similarity to compare to as this is the first-time representations learned from behavior have been quantitatively validated against propositions from the domain.

### Course prerequisite and degree requirement evaluation

American liberal arts universities, such as UC Berkeley, are known for their high degree of elective choice afforded to students [27, 28]. This permits students to choose from many courses in order to satisfy most university and degree-specific graduation requirements. It can therefore be hypothesized that the individual enrollment choices made within this high degree of freedom are an important signal that contributes to the information embedded in the learned course representation. An alternative hypothesis is that the majority of the informational signal comes from prerequisite and degree requirement structures governing student choice. If this were true, enrollment data, which are difficult to obtain, could be replaced with often public prerequisite and degree structure information. We evaluate the relative information communicated by prerequisites and degree requirements compared to the addition of enrollment choices by synthesizing course sequences sampled from these structures and re-learning course representations from the sampled sequences. These representations are then evaluated on our analogy and equivalencies validation sets and compared with the results from the enrollment-based representation on the same sets.

#### Prerequisite-based course sequence generation

There are 5,612 prerequisite course pairs in total at UC Berkeley. We denote the prerequisite course pairs as a tuple set *P* = {(*p*_1_, *t*_1_), (*p*_2_, *t*_2_), …, (*p*_*n*_, *t*_*n*_)}, where *p*_*i*_ is the prerequisite course of *t*_*i*_ and *n* denotes the total number of prerequisite course pairs. Here we take *p*_*i*_ as a prerequisite course and *t*_*i*_ as a target course. The goal is to generate all the possible longest prerequisite course sequences from *P* based on the rule that any course *i* in the sequence should be the prerequisite course of the course right after course *i*. Any generated sequence should not be a sub-sequence of any sequence in the generated sequences set. For instance, it is intuitive to construct all the sequences from the set {(*A*, *B*), (*A*, *C*), (*B*, *D*), (*C*, *D*), (*D*, *E*)}, which are [*A*, *B*, *D*, *E*] and [*A*, *C*, *D*, *E*]. Here, a sequence such as [*A*, *B*, *D*] should not be included in the final sequences set because it is a sub-sequence of [*A*, *B*, *D*, *E*]. When the number of prerequisite course pairs increases, the pairs in *P* constitute a large complex graph *G*. Exact enumeration of all the sequences from a large graph is NP-hard. An algorithm combining a random walk and skip-gram model [29] was therefore used to learn node embeddings from our prerequisite graph. A walk uniformly samples from the neighbors of the last vertex visited until the maximum length is reached to generate a sequence. Our approach differs from [29] in that: (1) We choose the start vertex to be a root prerequisite course that is a prerequisite for a course but does not itself have any prerequisites, as opposed to any arbitrary course serving as the start vertex (2) The end vertex of a prerequisite course sequence is a leaf course that has at least one prerequisite but is not itself a prerequisite, as opposed to a maximum length specification as was done in [29]. This process generated 4,001 sequences and learned course representations using the skip-gram model. Given that these sequences were different in length and number of unique courses from the real student dataset, a mild hyperparameter search of 20 random hyperparameter settings from the sets evaluated on real student data was conducted, keeping the best average scoring setting on the validation sets. There were 1,426 courses in the 4,001 generated sequences, compared with the 1,467 courses in the prerequisite courses graph.

#### Degree requirement-based course sequence generation

There are 223 undergraduate degrees at UC Berkeley, also known as academic plans listed in the dataset that drives the University’s Academic Guide [30]. There are 1,108 unique requirement categories and 7,317 courses satisfying one or more requirement categories in these data. The median number of requirement categories per degree is eight, with a median of four courses per requirement category as choices to satisfy the requirement. Our data regarding the structure of degrees did not contain information about the number of courses from each requirement category needed to satisfy the category, though notes in the Academic Guide suggest that this number is typically one or two. There is not a perfect match between these degree requirement data and our historic enrollment dataset, as some students in our historic dataset may have declared majors that are no longer offered and some departments have recently created new majors or courses for which we have no data.

To produce a dataset of synthesized student enrollment sequences generated purely from degree structure information, we iterated through each of the 223 degrees, randomly sampling two courses from each of the degree’s requirement categories and from the College-level breadth requirement categories, of which there were typically seven. Though there are normative orders in which students are advised to satisfy requirements, our degree requirements data do not encode a suggested ordering. This lack of ordering could negative affect the ability of a skip-gram model to pickup on signal from our generated sequences. Course prerequisites are a source of ordering information and were added to address this potential issue. If a randomly sampled course was a prerequisite of a course already added to the synthetic student’s sequence, the sampled course was placed immediately prior to that course in the sequence. After a single synthetic student’s enrollment sequence was generated for each of the 223 degrees, the process was repeated until the total number of enrollments in this synthetic dataset equalled or exceeded 2.1 million, approximately the number of enrollments in our real student historical data. A skip-gram model, using the same hyperparameters as the best real data model, was trained on these synthetic sequences to learn course representations.

#### Validation results of structure and enrollment informed representations

We first evaluate the validation set performance of the embedding learned from sequences generated from prerequisite structure. After filtering out courses from the equivalency and analogy validation sets that are not among the 1,467 in the model trained on prerequisite sequences, 415 equivalency course pairs remain along with 1,500 analogy completions. The accuracy of the skip-gram model trained on the prerequisite course sequences on the equivalency and the analogy validation sets is 4.58% and 8.8%, respectively. The accuracy of the skip-gram model trained on the student enrollment sequences, using the same reduced validation sets, is 7.47% on the equivalency and 46.4% on the analogy set. This translates to an improvement of 63% (equivalency) and 427% (analogy) using student enrollments over prerequisite structure.

We next evaluate the validation set performance of the embedding learned from sequences generated from degree requirement structure with the addition of prerequisite ordering information. After filtering out courses from the validation sets that are not in the model learned from these sequences, 229 equivalency course pairs remain along with 700 candidate analogy completions. The accuracy of the skip-gram model trained on these synthetic sequences is 8.30% on the course equivalency validation set and 9.29% on the analogy set. The accuracy of the skip-gram model trained on real student enrollment sequences, using the same reduced equivalency and analogy validation sets, is 10.48% and 46.71%, respectively. This translates to an improvement of 26.27% (equivalency) and 403% (analogy) using student enrollments over degree requirement structure with prerequisite ordering information. Comparing the prerequisite structure-only model on this same validation subset results in an equivalency set accuracy of 6.99% and analogy set accuracy of 9.71%. These results suggest that individual student course selections play a significant role in informing the course representations and convey a substantially higher amount of information about course relationships (i.e., analogy validation) and moderately more information about course similarities (i.e., equivalency validation) than do degree and prerequisite structures.

## Concept decompositions

The ability to perform vector arithmetic analogies suggests that distributed representation of concepts are encoded in this space [31, 32]. Vector spaces are subject to standard linear algebra techniques, including projections which can isolate a concept, such as the degree of gender bias encoded in a word [33]. Representing a concept as a vector and projecting a course onto it can, ideally, suggest the degree to which a course is comprised of that concept. In our analysis, the concepts are subject vectors created by taking the average of course vectors in the subject (i.e., centroid). Individual courses are then projected onto the concept vectors and the magnitude of incident with the concept vector is plotted.

**Fig 4:**
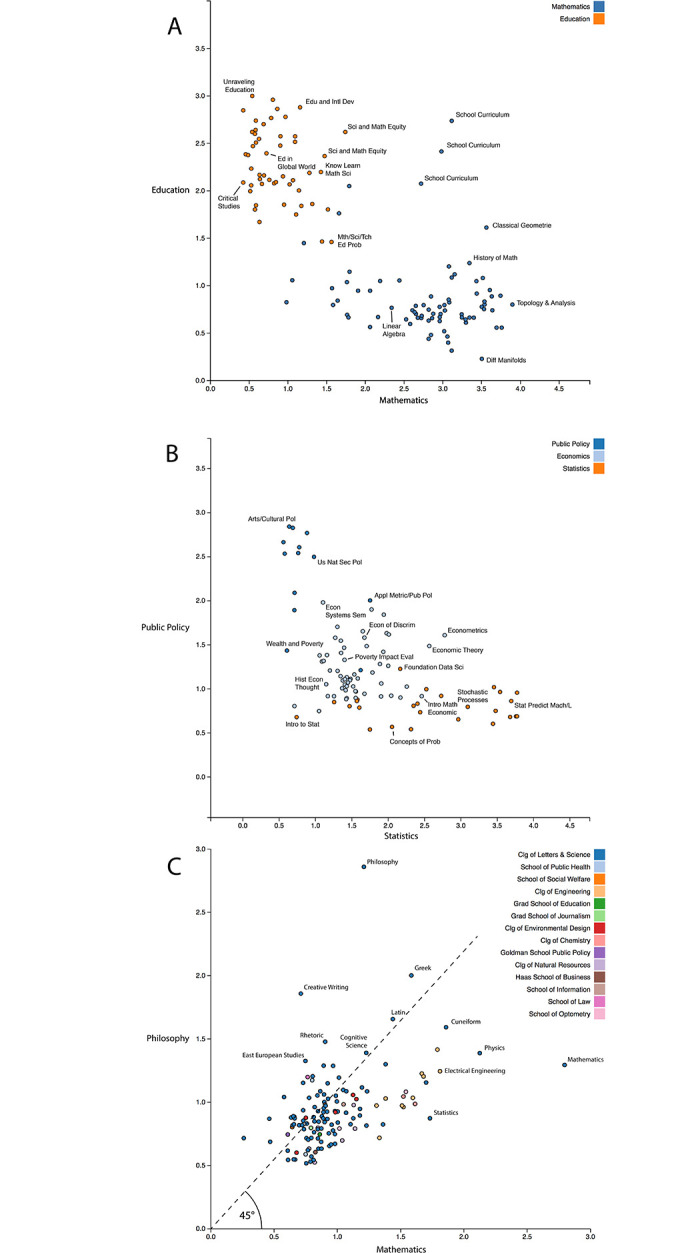
Conceptual decompositions. Conceptual decompositions of courses in the Subjects of (A) *Mathematics* and *Education*, (B) *Economics*, *Public Policy*, and *Statistics* and (C) all subject vectors.

We explored this approach on the subjects of *Mathematics* and *Education* and their respective courses (Fig 4A). Most of the courses stay high in their own subject magnitude (e.g., ‘Unraveling Education’ and ‘Topology & Analyses’ were highest in their respective subjects) and low in the other (e.g., ‘Critical Studies in Education’ and ‘Differential Manifolds’ were respectively lowest in their opposing subject). Certain courses are shown to be appropriately high in both subjects, such as a *Mathematics* course titled, ‘School Curriculum,’ taken by Mathematics majors with a teaching concentration and a course in *Education* titled, ‘Special Problems from Mathematics, Science and Technology Education’.

We also projected courses from *Economics*, a highly mathematical social science, onto *Statistics* and *Public Policy* as shown in Fig 4B. We find that the vector representations were able to capture the balance between Statistics and *Public Policy* well. Theory courses such as ‘Econometrics’, ‘Economic Theory’, and ‘Intro Math Economics’ were mapped high in the *Statistics* dimension, while courses such as ‘Economics of Discrimination’ were mapped highly in *Public Policy*. Courses regarding development studies and inequality closely mapped to the diagonal, suggesting relatively equal representation. Notably, the *Public Policy* course on ‘Applied Econometrics and Public Policy’ ranked the highest towards *Statistics* among *Public Policy* courses and ‘Foundations of Data Science’ ranked highest in *Public Policy* among *Statistics* courses.

We performed the same exploratory analysis on all subjects in the University, breaking them down in terms of their magnitude of *Mathematics* and *Philosophy* (Fig 4C). *Physics*, *Statistics*, and engineering subjects had the highest proportion of *Mathematics*, while *Creative Writing*, *Rhetoric*, and *East European Studies* had *Philosophy* in highest proportion (furthest from the 45° line). The subject of *Cuneiform*, an ancient system of writing, is arguably semantically misplaced. It appears high in *Mathematics* magnitude, though it is also moderately high in *Philosophy*.

In another exploration of the rationality of the subject vectors, we queried the embedding to describe a subject as a combination of two other subjects. The combination was calculated by adding element-wise, the two subjects’ vectors and finding the closest subject vector to this sum. Expectations for these subject combinations were not pre-defined, as the purpose of this experiment was exploratory, presenting the results for evaluation based on their face validity. A sampling of these results (Table 3) suggest that there are topical regularities encoded not only at the micro level of the embedding, shown in the course decompositions, but also more globally, as demonstrated by conceptually rational arithmetic closure at the subject level. Full pairwise subject composition results can be found in Supporting Information.

**Table 3:** Exemplar subject compositions.

Subject Compositions		
*Earth & Planetary Science* + *Physics*	→	*Astronomy*
*Asian Studies* + *Religious Studies*	→	*Buddhist Studies*
*Asian Studies* + *Classics*	→	*East Asian Languages*
*Business Admin* + *Statistics*	→	*Economics*
*Art Practice* + *History*	→	*History of Art*
*Business Admin* + *Computer Science*	→	*Information*
*Rhetoric* + *Political Science*	→	*Legal Studies*
*Health & Medical Sciences* + *Mathematics*	→	*Molecular & Cell Biology*
*Philosophy* + *Mathematics*	→	*Physics*
*Demography* + *Mathematics*	→	*Statistics*

## Visual mapping

**Fig 5:**
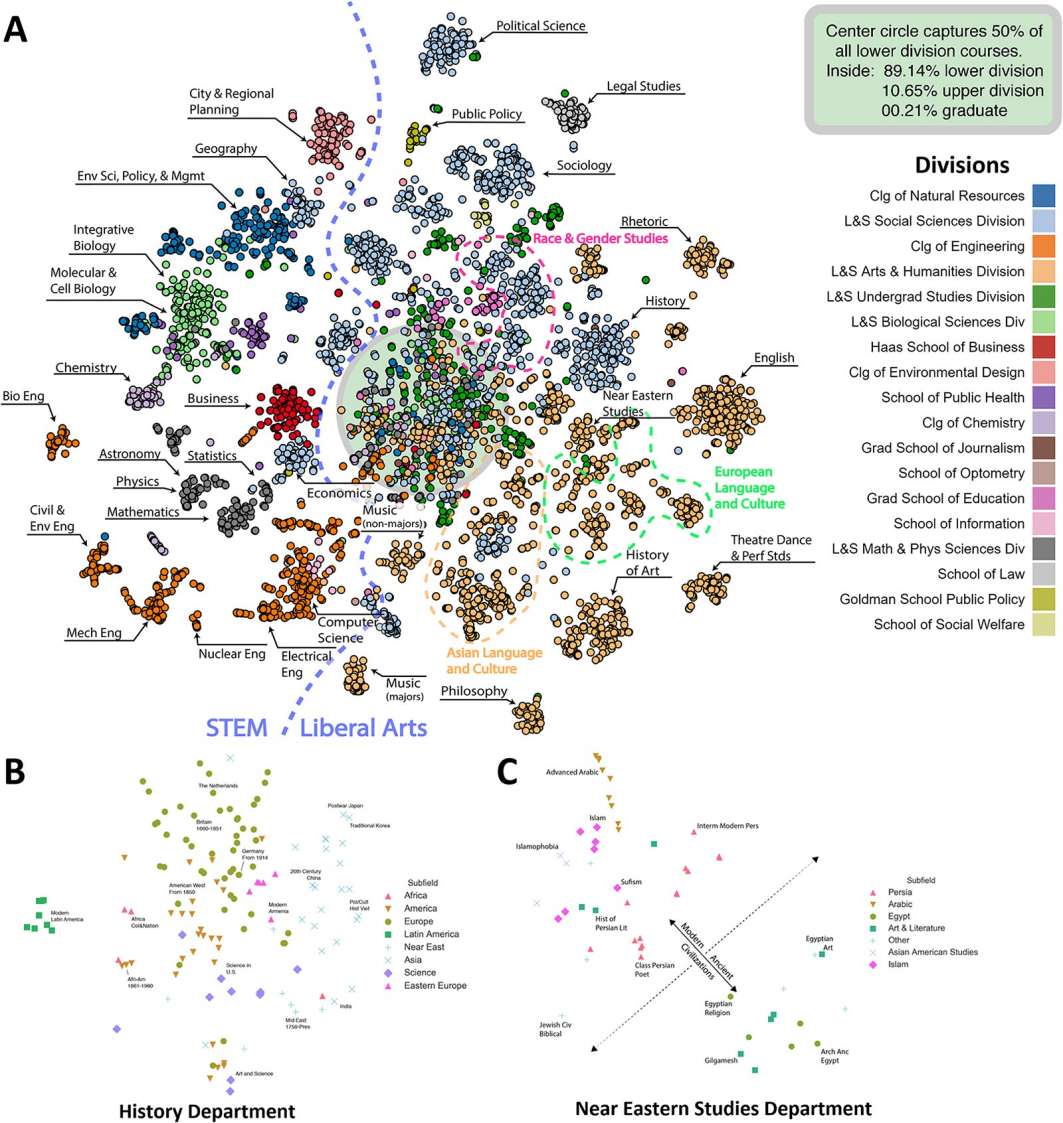
t-SNE 2-d projections. (A) all course vectors with close-ups of the departments of (B) *History* and (C) *Near Eastern Studies*.

We visualized the course embedding to surface the primary factors which dictate vector proximity in the space using Barnes-Hut t-SNE [34] for dimensionality reduction. This allowed for observation of micro, meso, and macro scale relationships not hypothesized and produced a first-of-its-kind view of the University and the relationships between its disciplines. Each data point in Fig 5A is a course, colored by the division it belongs to, with labels added for subject groupings. The t-SNE algorithm prioritizes the retention of local structure from the high-dimensional space in its manifold projection to the two-dimensional space (e.g., keeping data points close in the low-dimensional space that were close in the high-dimensional space) [35].

At the micro-level, the visualization reveals salient conceptual relationships between individual courses. Zooming into the History cluster, the courses organize roughly into a rotated map of the globe (Fig 5B). Starting at the top right are the East Asian countries: Japan and Korea with China to their west. Below them are Southeast Asian countries such as Vietnam and India to its west. Towards the west, we find Eastern Europe, Western Europe, and finally the United States, though some clusters do not adhere perfectly. This geographical layout can be explained by the tradition among historians to specialize in a time and a place with interest typically only extending to adjacent geographic regions and not to general themes that might cut-across disperse regions. Where the norms of the *History* department placed courses geographically, Near Eastern Studies separates them temporally, with a boundary between courses covering modern and ancient civilizations (Fig 5C). We find that ancient literature, religions, and societies such as Egypt, map towards the lower right whereas modern languages and religions such as Arabic and Islam, populate the top left, representing the discipline’s bi-modal foci.

**Fig 6:**
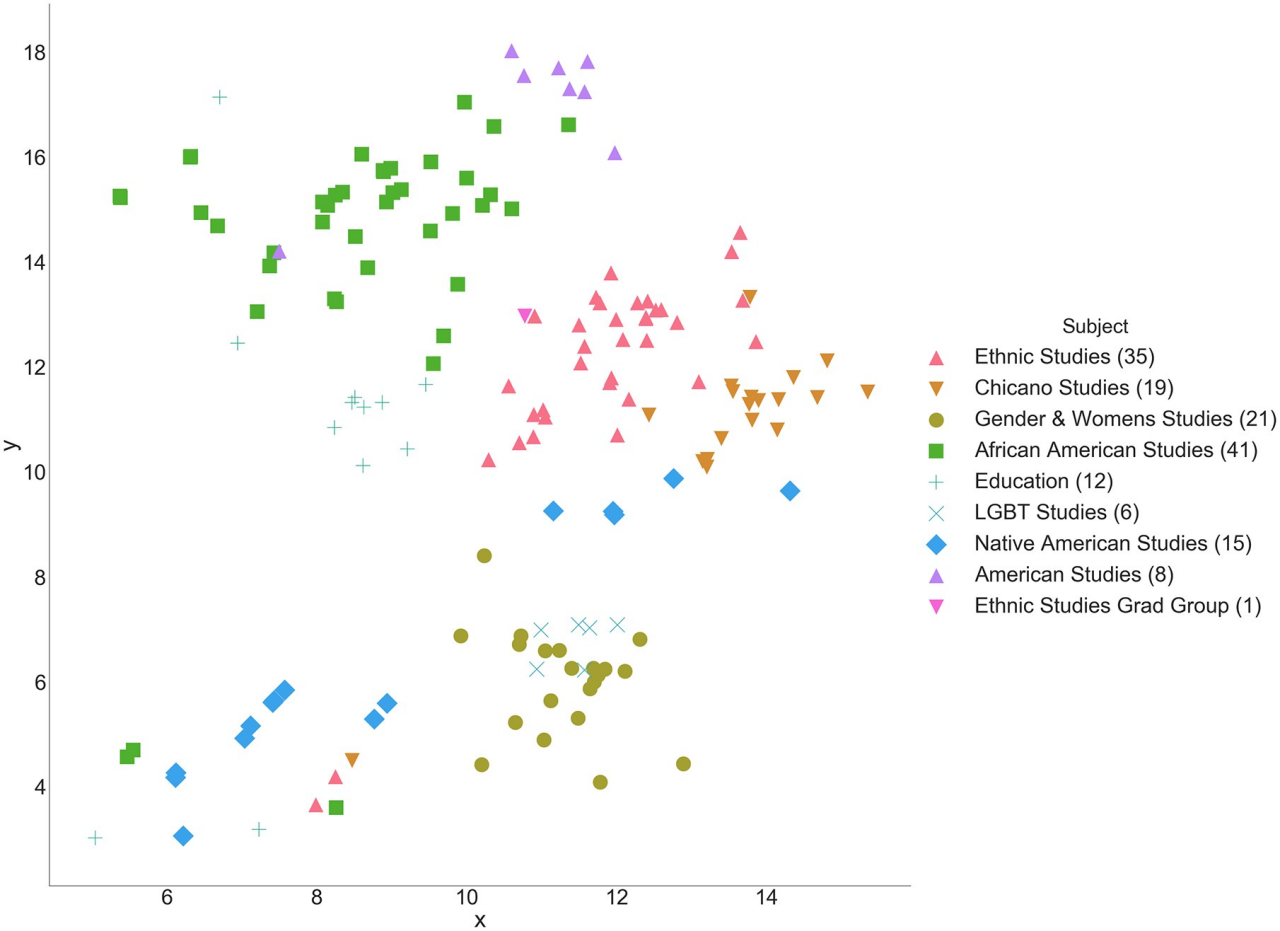
Close-up of race & gender studies cluster.

**Fig 7:**
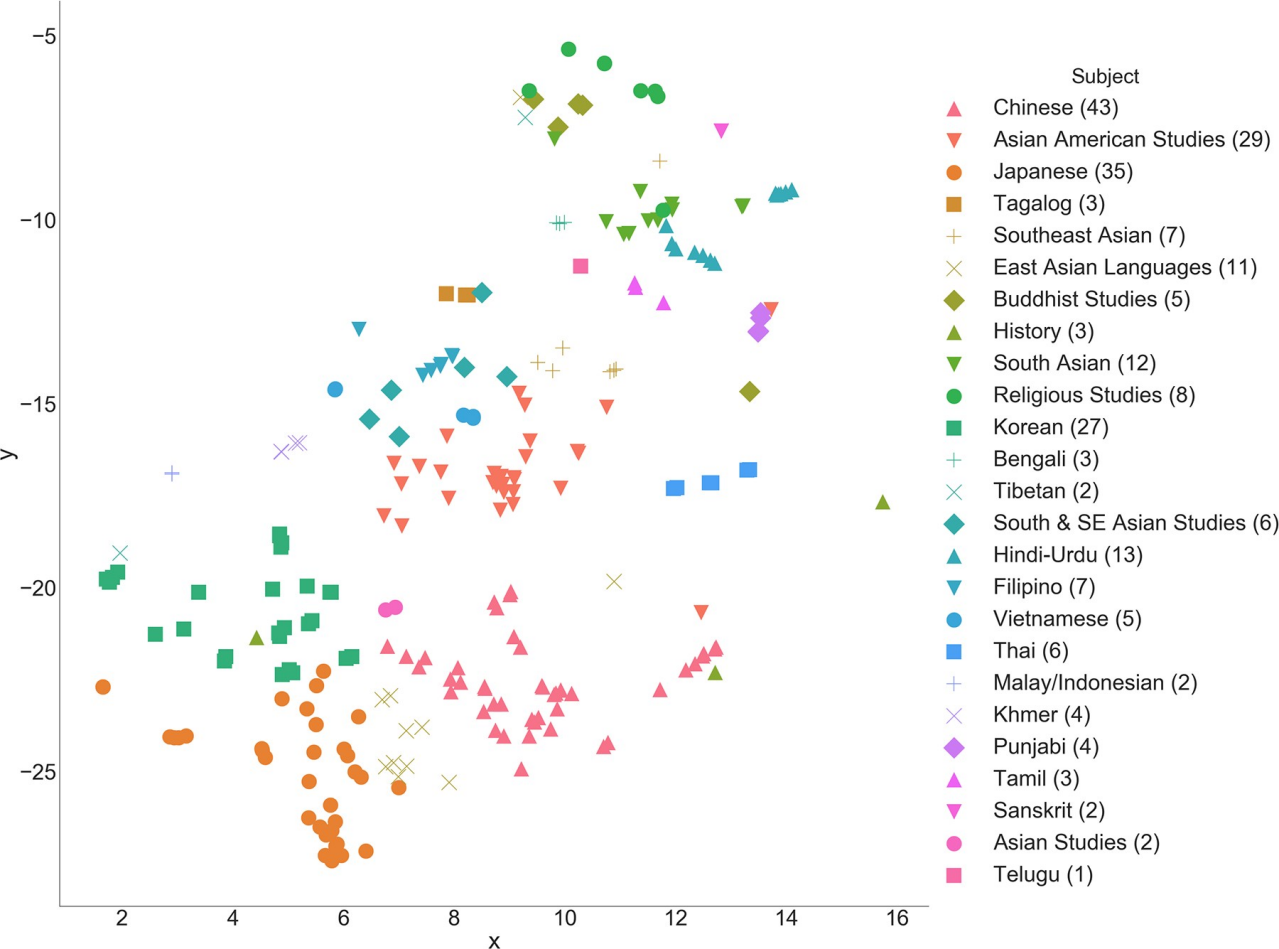
Close-up of Asian languages & culture cluster.

Logical meso-level relationships can also be seen, with *Statistics* situated between *Mathematics* and *Economics* and *Physics* between *Mathematics* and *Astronomy* (Fig 5A). An interesting path begins in *Chemistry*, traversing through *Molecular & Cell Biology*, *Integrative Biology*, *Environmental Science & Policy Management*, *Geography*, *City & Regional Planning*, and terminating at *Architecture*. The subjects progress with conceptual coherence between neighbors such that, though *Chemistry* and *Architecture* have little in common, the relationship between each intermediary subject is logical. This adjacency of disciplines naturally bears resemblance to relationships seen in the broader study of academic citation networks [36–38]. While the majority of courses group by subject and department, interdisciplinary groupings are observed in the thematic areas of Race & Gender Studies, European Language and Culture, and Asian Language and Culture (Figs 5A, 6 and 7).

A noticeable feature of the visualization is the unstructured cloud of largely Lower Division level courses near the origin, contrasted against the more structured clusters of Upper Division courses outside of it. Berkeley classifies Lower Division courses as part of the introductory sequence to an academic discipline often taken by prospective students of the associated major or to fulfill Colleges’ mandatory breadth requirements. Lower Division courses generate a high degree of variance in the enrollment contexts in which they appear due to being taken by a wide variety of majors. Because of this, they may exhibit fewer distinguishing regularities and structure, as opposed to Upper Division courses which often build on prior knowledge and are mostly taken by students within a major associated with the course’s department.

Finally, at the macro level, a bisection of the entire map divides subjects considered to be Science, Technology, Engineering, and Math (STEM) [39] on the left side from Liberal Arts subjects on the right. Courses offered by the College of Engineering reside close to the bottom left quadrant, Natural Sciences to the upper left, Social Sciences to the upper right, and Arts & Humanities to the bottom right. Departments under the Social Sciences Division are largely found in the Liberal Arts hemisphere with the exceptions of *Psychology* and *Economics*, both of which have highly statistical facets. Though the STEM classification of courses in our embedding is not new knowledge, it demonstrates that the embedding can capture information not likely fully known by any of the individuals whose actions it was produced from. This observation also underscores the impressive ability of t-SNE to render a single projection with conceptual coherence retained at several levels of scale.

The salience of clustering by subject in the t-SNE visualization begged the question of what other course attribute information was encoded in the higher-dimensional space. To quantify the information captured, we trained multinomial logistic regression models [40] using course vectors as the input to regress to six different categorical attributes kept by the Registrar’s Office and found in our enrollment metadata. This model performed well in predicting the attribute values of a held-out test set of courses, with the subject of a course predicted with 84.19% accuracy based on its vector compared to 3.01% when predicting using the most common subject. Overall, attribute values were predicted with 87.95% accuracy using the embedding compared to 30.63% by majority class (Table 4).

**Table 4:** Results of predicting attributes from course vectors.

Attribute	Unique Values	Majority	Logistic
Subject	197	3.01%	84.19%
Department	81	5.01%	87.06%
Division	20	27.15%	84.92%
College	16	64.61%	94.60%
Course Level	3	57.11%	91.06%
Modal Major	114	26.88%	85.86%
*Average*		30.63%	87.95%

## Semantic mapping

While rich in structure, the vector values of an embedding alone lack interpretability. In the previous sections, we added semantic meta information, such as course titles and subject membership to facilitate interpretation of relative course vector proximities as depicted visually with t-SNE and algebraically using subject vector centroids. The interpretations of *History* and *Near Eastern Studies* close-ups (Fig 5B and 5C) were facilitated by experts, identified in Acknowledgements, whose consultations effectively served as an addition of semantics to the space. In this section, we use course catalog descriptions, collected from Berkeley’s Course API and concatenated with course titles, to automatically add fine-grained semantics to the vector space, provided by an expert (i.e., the course creator). This semantic mapping of a vector space, which can be algebraically queried, can itself be seen as an expert informational representation, with epistemic expertise defined by the ability to justify knowledge with propositions in the domain [41]. This is in contrast to deep neural networks, where expertise is commonly demonstrated by the ability to perform [42–45]. Both types of neural-based experts have distributed representation at the core of their generalizing principle.

To map semantics onto the embedding, we trained a multinomial logistic regression mapping course vectors to their bag-of-words course descriptions sourced from the University course catalog. This was a neural machine translation, not between languages [46], but between a course representation space formed from behaviors and a semantic space constructed from instructors’ natural language descriptions of each course. This mapping allowed arbitrary vectors in the space to be semantically described using keywords, those words regularized by way of their regression from the feature space of the embedding. To control the level of specificity of the words output by the model, we introduced a bias parameter (Eq 3). A higher bias would result in less common and more distinct words that could be considered discipline-specific jargon, while a lower bias would produce descriptions using more common vocabulary. While we initially applied tf-idf [47], the brevity of course descriptions usually yielded at most one instance of each word in a description, effectively nullifying the term-frequency weight component and reducing tf-idf to only idf. Experimentally, we found that treating the entire collection of descriptions as one document and exponentiating the raw frequency to a negative number yielded a desirable spectrum of word specificity.

$$\begin{array}{c} tf-bias={\left(\frac{number\;of\;occurrences\;of\;word}{total\;word\;count}\right)}^{-bias} \end{array}$$ (3)

We removed stop-words, stemmed words using the snowball algorithm, and used iterative bi-gram phrase detection to tokenize phrases before collecting tokens into bag-of-words vectors. To remove overally general words and words related to course logistics, we filtered words across four different metrics, taking only the top 100 words in each and hand-selecting words to be excluded from the set (Table 5). We kept words that could be meaningful in certain contexts even if they could also be generic in other contexts. For example, the phrase ‘web site’ may in some contexts indicate logistics of course taught through an online modality, but could also be more distinctly relevant in subject areas such as design, media, and information, where ‘web site’ may describe part of the subject matter of the course.

**Table 5:** Rules for preprocessing the semantic model training corpus.

Target	Sorting Metric	Reason	Top Removed	Top Kept	Total Removed
Phrases	Number of occurrences	Common phrases are more likely to reflect logistics	Freshman sophomore seminar	Case study	68 phrases
Phrases with at least 20 occurrences	Number of words	Longer phrases are more likely to reflect logistics	Structure vocabulary cultural social appropriate context speaking listening ability development oral exercises class discussion recordings available Berkeley language center reading writing development class exercises independent reading project composition	African American Asian American	78 phrases
Words	Number of occurrences	Common words are more likely to be vague	Course	Development	51 words
Words	Number of subjects they appear in	Breadth of words suggest vagueness and logistics	Covered	Current	76 words

Using the final processed descriptions, we trained a multinomial logistic regression model where the course vectors were used as input features and their corresponding tf-bias processed course description words were used as outputs. We refer to this as the *semantic model*. We trained several models, varying tf-bias and max epoch values. Manual qualitative inspection of a sampling of trained models suggested that lower tf-bias and training epochs produced more general words whereas higher tf-bias and training epochs produced more specific words.

To explore the ability of the semantic model to resolve vectors from the embedding to sensible natural language semantics, we first ran subject centroid vectors through the model to see whether the highest probability output words appropriately described each subject. Table 6 compares biases 0.5 and 1 across three exemplar subjects. A bias of 0.5 preferred broader words such as “Algorithms”, “Markets”, and “Society” to describe the *Computer Science*, *Economics*, and *Sociology* vectors, respectively. Alternatively, a bias of 1 surfaced “Robotics”, “Game Theory”, and “Comparative perspective” as predicted words for those subjects. Particularly frequent descriptions appeared in both bias lists, such as “Computer”, “Industrial organization”, and “Inequality”.

**Table 6:** Semantic model descriptions of subject vectors using biases of 0.5 and 1.

*Computer Science*		*Economics*		*Sociology*	
0.5	1	0.5	1	0.5	1
Computer	Algorithms	Economic	Economic	Sociological	Sociological
Design	Computer	Theory	Industrial organization	Social	Inequality
Algorithms	Computer science	Analysis	Size	Inequality	Social change
Techniques	Program language	Determinants	Linear regression models	Social change	Social
Models	Implementation	Policy	Pricing	Society	Hypotheses
Control	Codes	Markets	Boom	Theory	Trends
Data	Machine	Development	Econometric	Institutions	Thought
Applications	Privacy	Pricing	Income	Thought	Dominant
Structure	Artificial intelligent	Industrial organization	Game theory	Trends	Comparative perspective
Project	Robotics	Size	Valuation	Within	European countries

We then queried the model to describe the vector centroids of three subjects (*Design Innovation*, *Neuroscience*, and *Plant Biology*) for which not a single course’s description from the subject was included in the training of the semantic model. The catalog descriptions of courses in these particular subjects were missing due to a limitation of the API used to access the catalog at the time, creating a naturally occurring opportunity for an experiment. *Neuroscience*, for example, produced words such as “brain”, “physiology”, “sensory”, and “neuroanatomy,” words likely borrowed from other subjects in biology. *Design Innovation* produced apt words such as “team”, “user”, “technology”, “interface”, and “robotics.” These descriptions, in Table 7, demonstrate the model’s ability to interpolate semantic meaning across sparse regions of the space.

**Table 7:** Semantic model description of missing subjects, the origin vector, and course vector differences (0.5 bias).

*Design Innovation*	*Neuroscience*	*Plant Biology*	Origin Vector	ECON 141—ECON 140	MATH H113—MATH 113	ARTHIST 32—JAPAN 1A
Team	Brain	Microbial	Cultural	Variants	Enjoy	Tumuli
Enable	Human brain	Molecular	History	Vector	Hidden	Seventeenth
User	Physiology	Preservation	World	Theorem	Hard	Newcomers
Share	Neurological	Plant	Social	Mathematical	Beauty	Neolithic
Innovation	Sensory	Biotechnology	Development	Quadratic forms	Corresponding	Nineteenth century
Perception	Biology science	Habitat	Society	Eigenvectors	Recommended	Proceed
Technology	Neural	Metabolic	Language	Discrete continuing	Honors	Art architecture
Interface	Neuroanatomy	Genomics	Political	Integer	Rigorous	Chronological
Robotics	Neurophysiology	Genetics	Modern	Function complex variables	Inclination	Focus particular
Vision	Anatomy	Biology	Human	Conditions expected	Greater	Realism

An emergent [48] set of regularities highlighted by the course analogies were the vector offsets between two courses which represented a relationship (e.g., vec[Honors Linear Algebra]—vec[Linear Algebra]). The accuracy of the course analogies, and patterns observed in the space (Fig 3), suggest that these vector offsets are themselves representative of a shared distributed concept or regularity. We used the trained semantic model to attempt to describe these vector offsets. For instance, subtracting *Japanese* 1A (‘Elementary Japanese’) from *History of Art* 32 (‘Art and Architecture of Japan’) produced a vector described by the model as, “tumuli”, “Neolithic”, “art-architecture”, and “realism,” words appropriate for describing art history. While we ascribed the relationship between *Economics* 141 and 140 as a more mathematically “rigorous” treatment of Econometrics, the semantic model succeeded in articulating granular topical differences, using words like “vectors,” “discrete-continuous,” and “conditional expectations” to accurately describe the content that is in 141 but not in 140. This offset vector, produced by subtracting ECON 140 from ECON 141, had two Linear Algebra courses, MATH 110 and MATH 113, as its nearest neighbors. Other words that appeared, such as “quadratic forms” and “eigenvectors,” while not explicitly taught as part of the course material, are related to linear algebra, the topic only found in the more advanced offering (Table 7). The semantic model, leveraging regularities formed from enrollment choices, surfaced topical differences not found in either course’s catalog description. The original descriptions are shown below with words underlined that are not shared between the two descriptions:

**Economics 140**: Introduction to problems of observation, estimation, and hypothesis testing in economics. This course covers the linear regression model and its application to empirical problems in economics.
**Economics 141**: Introduction to problems of observation, estimation, and hypothesis testing in economics. This course covers the statistical theory for the linear regression model and its variants, with examples from empirical economics.

The ability to describe any arbitrary vector allows for queries that have no correspondence to a particular course, but are conceptually interesting nonetheless. The origin vector (i.e., all zero vector) could be interpreted as the center of UC Berkeley’s academic, liberal arts demography, but otherwise has no educational meaning. The semantic model describes the origin with the words “cultural,” “history,” “world,” “social,” and “development” as the top five results.

## Discussion

Visualization of the course embedding at several scales evokes images of cell-cultures in a petri dish under a microscope or a deep field view of constellations through a telescope. Our domain of study can be viewed analogically as elements—courses—introduced into the social system of a university with human factors serving as the forces dictating the movement of the elements and their positionality in the structure as a whole. This representational structure, illuminated by data and studied through the instrument of a learned embedding analysis, is analogous to the physical structures studied with instruments from the natural sciences and is part of a larger universe of explorable structure expanding at the speed of data collection. A question of natural concern to the developing notion of data science is whether truths can be learned from behavioral data through this particular lens of a representation analysis. Our study used a variety of inference types to interrogate the embedding for such truths: abductive inference to describe patterns in the visual mapping, inductive inference to define subjects by an aggregation of their courses using concept decompositions, and deductive inference to validate analogies (i.e., by syllogism). If known truths about courses were to be defined exclusively as the instructors’ catalog descriptions, then the semantic interpolation was able to successfully surface previously unknown truths about the topics of courses with no catalog description in the data and about topical differences between courses not found in their descriptions. We conclude that considerable knowledge is made accessible using these methodologies from representational structure formed by enrollment histories; with the validity of individual inferences dependent on the veracity of the regularities, known to increase with data volume. It is expected that when applied to other data contexts, semantics about elements truly unknown to a domain could be revealed.

A corpus is considered to be unstructured data in computational fields. This is not to discount that there are well known structures which guide production of natural language in a corpus. Grammatical structures provide a base level of constraint, on top of which social structures govern the topics which are discussed and the norms of how they are discussed. Finally, individual preferences and expression of an author, and perhaps editor and reviewer, ultimately decides what is written. This is not unlike our dataset of unstructured enrollment histories. Degree requirements and prerequisites provide a grammar-like constraint, on top of which social norms for course taking may be informed by peers and incentivised by employers and graduate schools. At universities in the United States, it is ultimately the student’s individual preferences, given what is available and advisers’ reviewer-like suggestions, that decide which courses will be taken. In both the language and university domain, much is already known about the structures which guide behavior. Our work demonstrates that more can be described about a domain from by studying behaviors within these structures than by studying the structures alone. The types of structures (e.g., rules, policies, and constraints) that allow for desirable regularities to form out of behavior is a topic for future work.

Data science methodologies will continue to advance in their ability to faithfully derive structure from unstructured data. Neural language models based on contextual embeddings (e.g., BERT [9]) have shown promise in their ability to perform well at prediction tasks, with nascent work showing evidence of capturing syntactic structure [49] and linearities in the sub-spaces of these deep models [50, 51] that may make them amenable to the more epistemic evaluation and semantic exploration performed in this paper. Domains that may most benefit from structure learning approaches (i.e., neural embeddings) are those in which unstructured data is all that has been observed, or where the structures governing production of the data are scarcely observed or understood.

An embedding learned from behavioral data may encode attributes and aggregated tacit knowledge by mechanisms such as the wisdom of crowds [52, 53], distributed cognition [54], or the combination of expert opinions [55] or classifiers [56, 57]. However, like the cultural biases reflected in word embeddings [33, 58], a course embedding too has an anthropological epistemology. It is perhaps most aptly characterized as students’ perceptions of courses at the time of enrollment, influenced by peer testimonials and degree requirements (faculties’ representations of their relatedness). With social-behavioral data, the embedding, and data science itself, takes on a dual identity of aiding in the pursuit of truths on one hand and on the other, reflecting the disposition of the individuals and society whose data it is constructed from.

Since this work began [59], the course vector representations have been integrated into a campus course recommendation system [17], allowing students to explore courses with conceptual overlap with a favorite course of theirs [60]. The semantic mapping technique has been used to augment course catalog descriptions with searchable inferred course topics [61], and translation between two institutions’ course vector spaces has been shown to be capable of surfacing academically equivalent courses to expand transfer student pathways, a processes known as course articulation [62].

## Supporting information

S1 File (ZIP)

S1 Data (TXT)
